# Association of the VDR rs1544410 Polymorphism with Metabolic Syndrome and Cardiometabolic Traits in Institutionalized Older Adults

**DOI:** 10.3390/ijms27125212

**Published:** 2026-06-09

**Authors:** Szymon Michniewicz, Krzysztof Chmielowiec, Magdalena Gibas-Dorna, Bartłomiej Czyżniewski, Ewa Pruszyńska-Oszmałek, Paweł Kołodziejski, Michał Tomasz Kowalski, Anna Grzywacz, Jolanta Chmielowiec

**Affiliations:** 1 Department of Humanization of Health Care and Sexology, Collegium Medicum, University of Zielona Góra, 28 Zyty Street, 65-046 Zielona Gora, Poland; s.michniewicz@inz.uz.zgora.pl; 2 Department of Hygiene and Epidemiology, Collegium Medicum, University of Zielona Gora, 28 Zyty Street, 65-046 Zielona Gora, Poland; k.chmielowiec@inz.uz.zgora.pl; 3 Department of Anatomy and Histology, Collegium Medicum, University of Zielona Gora, 28 Zyty Street, 65-046 Zielona Gora, Poland; m.gibas-dorna@inz.uz.zgora.pl (M.G.-D.); b.czyzniewski@inz.uz.zgora.pl (B.C.); 4 Department of Animal Physiology, Biochemistry and Biostructure, Poznan University of Life Sciences, 35 Wołynska Street, 60-637 Poznan, Poland; ewa.pruszynska@up.poznan.pl (E.P.-O.); paw-el.kolodziejski@up.poznan.pl (P.K.); 5 Clinical Department of Cardiology, Multispecialty Hospital in Nowa Sol, 7 Chalubinskiego Street, 67-100 Nowa Sol, Poland; kowaltmd@wp.pl; 6 Independent Laboratory of Genetics and Behavioral Epigenetics, Pomeranian Medical University in Szczecin, Powstancow Wielkopolskich 72 Street, 70-111 Szczecin, Poland; grzywacz.anna.m@gmail.com

**Keywords:** metabolic syndrome, VDR rs1544410, triglycerides, NEFA, elderly, anthropometrics, gene-MetS interaction

## Abstract

The rs1544410 (BsmI) polymorphism of the vitamin D receptor (VDR) gene has been implicated in metabolic regulation, although its role in metabolic syndrome (MetS) and related phenotypes remains unclear. This study aimed to evaluate associations between rs1544410, MetS status, and anthropometric and biochemical parameters in institutionalized older adults. A total of 95 participants were included, of whom 40% met the criteria for MetS. Anthropometric and biochemical profiles were assessed, and rs1544410 genotyping was performed. Differences between MetS and non-MetS groups were analyzed, and two-way ANOVA was used to evaluate genotype, MetS status, and their interaction effects. Participants with MetS showed an adverse cardiometabolic profile, characterized by higher triglycerides (TGs), waist-to-hip ratio (WHR), and atherogenic index of plasma (AIP), as well as lower HDL-C levels compared with non-MetS individuals. No differences were observed for total cholesterol (TC), LDL-C, or non-esterified fatty acids (NEFAs) between groups. Genotype distributions did not differ between MetS and non-MetS participants. However, significant genotype × MetS interactions were observed for TG and NEFA, with a borderline interaction for WHR that was not confirmed in post hoc analyses. Carriers of the rs1544410 AA genotype within the MetS group exhibited higher TG and NEFA levels compared with other genotypes, whereas no genotype-dependent differences were observed in the non-MetS group. Importantly, AIP was higher in participants with MetS, with the highest values observed in AA genotype carriers. In conclusion, the rs1544410 polymorphism was not associated with MetS status but was linked to MetS-related differences in TG, NEFA, and AIP, suggesting selective effects on lipid metabolism.

## 1. Introduction

Metabolic syndrome (MetS) is a heterogeneous, multifactorial clinical entity characterized by the coexistence of several metabolic abnormalities that substantially increase the risk of cardiovascular disease and type 2 diabetes [1]. MetS is commonly defined as a cluster of interrelated metabolic and anthropometric disturbances, including central obesity, dyslipidemia, elevated blood pressure, and impaired glucose metabolism [1,2]. This definition reflects the multifactorial nature of MetS and its relevance to cardiometabolic risk.

MetS is highly prevalent in older adults and contributes significantly to cardiovascular morbidity in this population. Epidemiological studies indicate that the prevalence of MetS increases progressively with age and varies depending on the diagnostic criteria applied, reaching particularly high levels in elderly populations [3,4,5]. In institutionalized older adults, reported prevalence rates vary widely across studies and regions, ranging from approximately 20% to nearly 60% [6]. Its occurrence is partly driven by aging-related metabolic dysregulation, including increased insulin resistance, altered lipid metabolism, and a higher prevalence of central adiposity [3]. Recent evidence also suggests substantial heterogeneity in metabolic profiles among older individuals, reflecting the complex interactions between biological aging, comorbid conditions, and functional decline [7]. Institutionalized older adults constitute a particularly vulnerable subgroup due to a high burden of multimorbidity, functional impairment, and increased dependence on long-term care. In this setting, metabolic disorders often coexist with frailty and polypharmacy, which may further exacerbate cardiometabolic risk [4,5].

Vitamin D (VD) has recently gained attention as a potential hormonal regulator involved in metabolic homeostasis. Epidemiological studies, particularly in older populations, have reported associations between higher circulating vitamin D levels and more favorable cardiometabolic profiles [8,9], whereas vitamin D deficiency is more frequently observed in individuals with MetS and its components [10,11]. However, the available evidence remains inconsistent, and the mechanisms linking vitamin D status to metabolic dysregulation, especially in aging populations, are not yet fully understood.

The biological effects of vitamin D are primarily mediated by the vitamin D receptor (VDR), a ligand-activated transcription factor encoded by the VDR gene and expressed in multiple metabolically relevant tissues, including adipose tissue, skeletal muscle, liver, and pancreatic β cells. Its wide tissue distribution underscores the systemic nature of vitamin D signaling, extending beyond its classical role in calcium and bone metabolism. Upon ligand binding, VDR forms a heterodimer with the retinoid X receptor and regulates gene transcription via vitamin D response elements, thereby modulating pathways involved in immune function, metabolic regulation, and cellular homeostasis [12]. In addition, non-genomic signaling mechanisms mediated by membrane-associated receptor forms have been described [13]. Notably, aging is associated with reduced VDR expression [14], which may contribute to increased susceptibility to metabolic disturbances [15]. In this context, both age-related changes in receptor expression and genetic variability within the VDR gene may play a role in modulating individual metabolic risk.

Given the central role of VDR in vitamin D signaling, polymorphisms within the VDR gene are considered potential determinants of inter-individual differences in metabolic responses [16]. Such variants may influence receptor activity and downstream signaling efficiency, thereby modifying metabolic processes. In particular, the rs1544410 (BsmI) polymorphism has been widely investigated in relation to metabolic traits; however, the findings remain inconsistent [17]. Moreover, data on the interaction between VDR polymorphisms and metabolic syndrome status, particularly in older institutionalized populations, remain limited.

The aim of the present study was to investigate whether the rs1544410 (BsmI) polymorphism of the VDR gene modifies metabolic and anthropometric characteristics according to MetS status in older individuals residing in long-term-care facilities. We hypothesized that VDR genetic variability interacts with metabolic status, contributing to differences in biochemical and anthropometric profiles.

## 2. Results

The study population consisted of 95 institutionalized elderly participants. Detailed descriptive statistics for all analyzed parameters are presented in Table 1.

On average, BMI values were within the overweight range, and a considerable proportion of participants met the criteria for abdominal obesity according to sex-specific waist circumference cut-offs (Table 2). The lipid profile was characterized by features consistent with atherogenic dyslipidemia, including elevated TG and reduced HDL-C concentrations. 

MetS was identified in 40.0% of participants, while the remaining individuals did not meet the diagnostic criteria. The most frequently observed abnormality was non-HDL cholesterol meeting the defined threshold or lipid-lowering treatment, followed by increased waist circumference and elevated blood pressure. Impaired fasting glucose or glucose-lowering treatment was less frequent. The distribution of MetS components and diagnostic criteria is presented in Table 2.

Comparison of clinical and biochemical parameters between participants with and without MetS is presented in Table 3.

Participants with MetS exhibited an adverse cardiometabolic profile. Specifically, significantly higher TG levels and WHR, together with significantly lower HDL-C concentrations, were observed in the MetS group (*p* < 0.001 for TG and HDL-C; *p* = 0.044 for WHR). Additionally, a significantly higher AIP was found in participants with MetS (*p* < 0.001). Fasting glucose, body weight, and waist circumference showed higher mean values in the MetS group, although these differences were not statistically significant. No significant differences were observed for TC, LDL-C, non-HDL-C, hip circumference, or NEFA (*p* > 0.05).

To assess the genetic background of the study population, rs1544410 (BsmI) genotype distributions were first evaluated for deviation from Hardy–Weinberg equilibrium (HWE). No significant deviation from HWE was observed in either the MetS or non-MetS group (Table 4), indicating that the observed genotype frequencies were consistent with expected population distributions. Subsequently, genotype and allele frequencies were compared between participants with and without MetS. No significant differences were observed in either genotype distribution (χ^2^ = 0.450, *p* = 0.451) or allele frequencies (χ^2^ = 0.406, *p* = 0.524) between groups (Table 5).

The effects of metabolic syndrome status, rs1544410 (BsmI) polymorphism, and their interaction on anthropometric and biochemical parameters were assessed using two-way ANOVA (Table 6).

Two-way ANOVA revealed significant effects of metabolic syndrome status on TG (*p* < 0.001), HDL-C (*p* < 0.001), NEFA (*p* = 0.003), and WHR (*p* = 0.006). No significant effects were observed for most anthropometric variables or LDL-C, with borderline effects noted for waist circumference (*p* = 0.055) and TC (*p* = 0.055).

Regarding rs1544410 (BsmI), significant associations were detected for TG (*p* < 0.001), NEFA (*p* = 0.007), and WHR (*p* = 0.036), whereas other parameters showed no genotype-related differences.

Significant MetS × genotype interactions were identified for TG (*p* < 0.001), NEFA (*p* = 0.049), and thigh circumference (*p* = 0.046), with a borderline interaction observed for TC (*p* = 0.056).

Multiple linear regression analysis showed that the AA genotype of the rs1544410 VDR polymorphism was associated with higher AIP values compared with the reference AG genotype (β = 0.143, *p* = 0.044).

**Figure 1:**
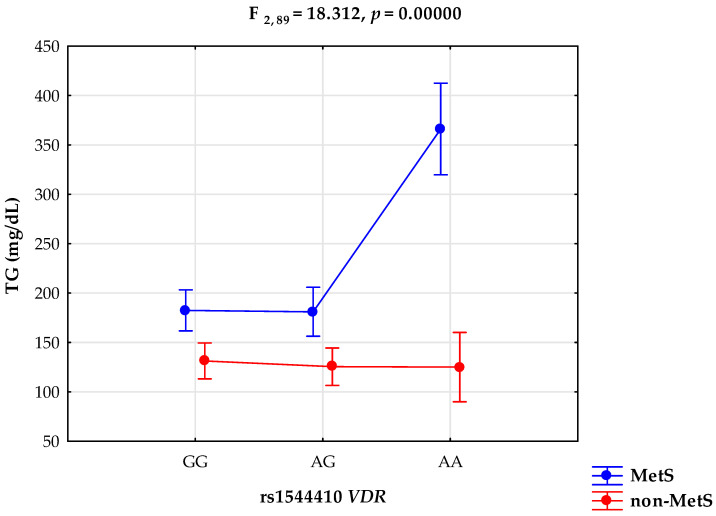
Interaction between VDR rs1544410 (BsmI) genotype and metabolic syndrome status on triglycerides (TG). MetS—participants with metabolic syndrome; non-MetS—participants without metabolic syndrome.

**Figure 2:**
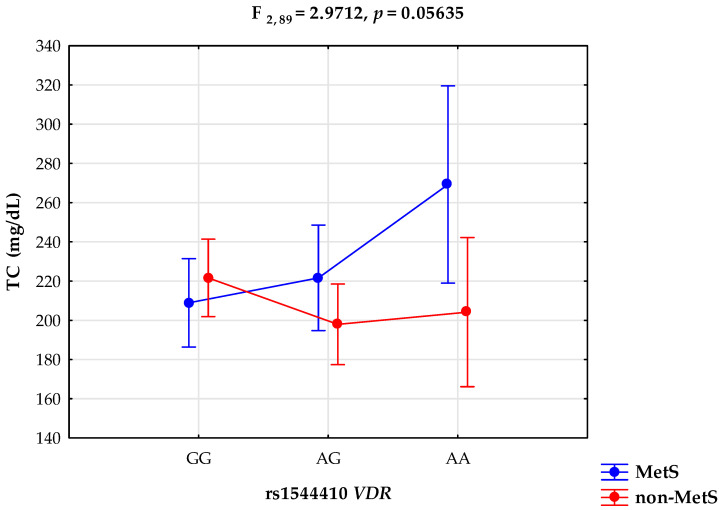
Interaction between VDR rs1544410 (BsmI) genotype and metabolic syndrome status on total cholesterol (TC). MetS—participants with metabolic syndrome; non-MetS—participants without metabolic syndrome.

**Figure 3:**
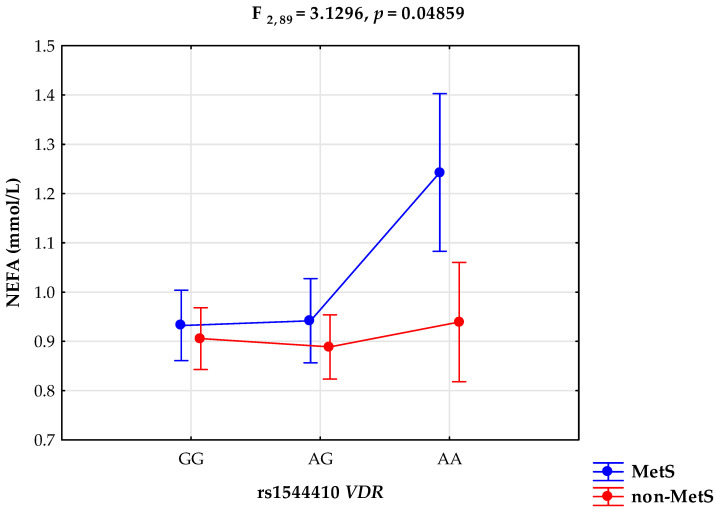
Interaction between VDR rs1544410 (BsmI) genotype and metabolic syndrome status on non-esterified fatty acids (NEFA). MetS—participants with metabolic syndrome; non-MetS—participants without metabolic syndrome.

**Figure 4:**
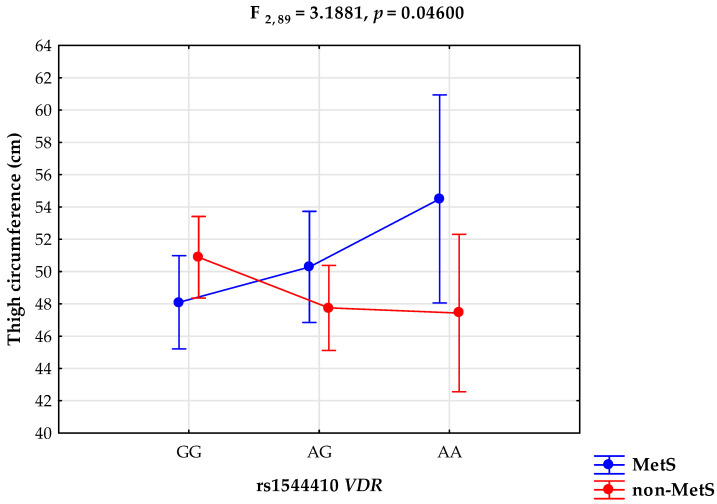
Interaction between VDR rs1544410 (BsmI) genotype and metabolic syndrome status on thigh circumference (cm). MetS—participants with metabolic syndrome; non-MetS—participants without metabolic syndrome.

**Figure 5:**
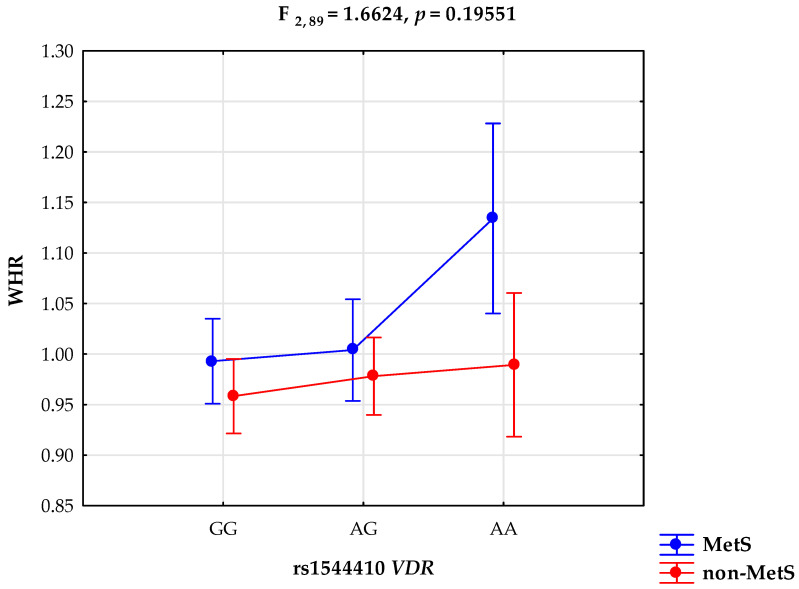
Interaction between VDR rs1544410 (BsmI) genotype and metabolic syndrome status on waist-to-hip ratio (WHR). MetS—participants with metabolic syndrome; non-MetS—participants without metabolic syndrome.

The interaction plots (Figure 1, Figure 2, Figure 3, Figure 4 and Figure 5) illustrate genotype-dependent patterns across MetS status for TG, TC, NEFA, thigh circumference, and WHR. Clear differences between MetS and non-MetS groups are most evident for TG and NEFA, particularly in AA homozygotes, where higher values are observed compared with other genotypes. For TC and WHR, more moderate genotype-related trends are visible across metabolic groups. Thigh circumference shows minimal variation across both genotype and metabolic status, with largely overlapping distributions.

Post hoc pairwise comparisons (Table 7) were performed to identify specific differences between rs1544410 (BsmI) genotype × metabolic status combinations.

AA homozygotes in the MetS group showed higher TG levels compared with all other groups (all *p* < 0.001), with no differences observed within the non-MetS subgroup. A similar pattern was observed for NEFA, with elevated values in AA carriers within the MetS group compared with both GG and AG genotypes as well as all non-MetS combinations (*p* ≤ 0.003).

For WHR, differences were mainly driven by comparisons involving AA homozygotes in the MetS group (*p* ≤ 0.017). For TC, only isolated significant differences were detected, mainly involving AA carriers (*p* = 0.043), while most comparisons were not significant. No significant differences were observed for thigh circumference after correction for multiple testing.

## 3. Discussion

Previous reports have linked the rs1544410 (BsmI) polymorphism of the VDR gene to metabolic alterations, including insulin resistance, and changes in lipid parameters that constitute diagnostic criteria of MetS [18,19]. In the present study, the rs1544410 polymorphism was not associated with MetS status in institutionalized older adults, as genotype and allele distributions were comparable between participants with and without MetS. However, significant genotype × MetS interaction effects were observed for TG, NEFA, and, to a lesser extent, WHR, indicating that the influence of VDR genetic variation becomes evident primarily under conditions of metabolic disturbance.

In particular, carriers of the AA genotype within the MetS subgroup exhibited significantly higher TG concentrations compared with GG and AG individuals. A similar pattern was observed for NEFA, where elevated levels were restricted to AA homozygotes in the MetS group. Importantly, multiple regression analysis demonstrated that the rs1544410 AA genotype was associated with higher AIP, suggesting that VDR genetic variation may modulate atherogenic lipid risk beyond the effects observed in individual lipid parameters. Nevertheless, given the limited number of AA homozygotes, these genotype-specific associations require confirmation in larger cohorts. In contrast, TC, LDL-C, and HDL-C did not show genotype-dependent differences, suggesting a selective effect of rs1544410 on TG-rich lipoprotein metabolism rather than a broad influence on lipid homeostasis. These findings are generally in agreement with previous studies reporting associations between VDR polymorphisms and lipid parameters, particularly TG and HDL-C; however, most available data do not account for metabolic syndrome status, which may contribute to differences in the observed effect patterns [20,21,22].

From a mechanistic perspective, the vitamin D receptor acts as a nuclear transcription factor regulating genes involved in extra-skeletal actions of vitamin D, including lipid and glucose metabolism, inflammatory pathways, and adipocyte function. VDR signaling influences insulin sensitivity and fatty acid metabolism through regulation of lipid uptake, storage, and oxidation pathways [23,24]. Although rs1544410 is an intronic polymorphism, it has been associated with altered VDR mRNA stability and gene expression, which may modulate downstream metabolic effects [25,26]. This may explain the genotype-specific differences observed in TG and NEFA, particularly under metabolic stress conditions.

Vitamin D status may further modulate these associations. Because serum 25-hydroxyvitamin D (25(OH)D) was not measured in the present study, direct assessment of gene–vitamin D interactions was not possible. Previous analyses in the same institutionalized cohort have demonstrated a high prevalence of vitamin D deficiency [27]. Reduced ligand availability may attenuate VDR signaling and enhance the relative impact of genetic variation. Experimental and clinical evidence suggests that gene–vitamin D interactions may be more pronounced under deficiency states [28,29,30], although confirmation in larger cohorts, including vulnerable populations, is still required.

The study population consisted of institutionalized older adults, a group characterized by a high burden of multimorbidity and metabolic disturbances. The high prevalence of MetS in the present cohort (40%) is in line with previous reports indicating a substantial cardiometabolic load in elderly care populations [31,32]. In this context, MetS represents an important clinical phenotype in aging, as it contributes to frailty, cardiovascular risk, and loss of functional independence. The relatively homogeneous living conditions in institutional care may reduce environmental variability, potentially enhancing the detection of genotype–phenotype associations, but at the same time limiting generalizability to community-dwelling populations.

The lipid profile confirmed an atherogenic pattern in participants with MetS, characterized by elevated TG and reduced HDL-C concentrations, reflecting established features of atherogenic dyslipidemia and increased cardiovascular risk [33]. Accordingly, AIP was higher in the MetS group compared with non-MetS individuals, reflecting a more atherogenic lipid profile. Based on established cut-off criteria for AIP [34], results in the MetS group indicated an elevated cardiovascular risk category. Beyond this classification, AIP reflects the combined effect of TG-rich lipoproteins and HDL metabolism that is not fully captured by individual lipid parameters. In line with this, previous studies have suggested that AIP may improve cardiovascular risk stratification by reflecting residual lipid-related risk beyond conventional lipid measures [35].

The very high prevalence of non-HDL-C abnormality observed in the present study likely reflects the specific characteristics of this institutionalized older adult population rather than the applied diagnostic thresholds alone. As previously demonstrated in analyses of the same cohort [27], participants exhibited a positive energy balance and an unbalanced macronutrient intake characterized by high fat and carbohydrate consumption. Low physical activity and the use of psychotropic medications, including atypical neuroleptics known to affect lipid metabolism and body weight, may have further contributed to the observed atherogenic lipid profile.

Importantly, rs1544410-related effects were mainly observed in biochemical traits, whereas most anthropometric measures (BMI, waist circumference, and hip circumference) showed no consistent genotype-dependent variation. A borderline interaction was observed for WHR, which was not confirmed in post hoc analyses, indicating a lack of a robust association for this parameter. These findings support the notion that VDR-related genetic variability is more closely linked to metabolic regulation than to body size or fat distribution phenotypes, which is supported by most previous studies showing no association between rs1544410 and anthropometric traits [28,36,37,38].

Several limitations of this study should be acknowledged. The sample size was modest, particularly for the AA genotype subgroup, which may limit statistical power. Consequently, some subgroup and interaction analyses may have been underpowered and should therefore be interpreted with caution. The cross-sectional design precludes causal inference, and the single-center institutional setting limits generalizability. In addition, serum 25(OH)D concentrations were not assessed in the present study, which precluded the direct assessment of gene–ligand interactions, despite previously reported high prevalence of vitamin D deficiency in this cohort. Nevertheless, the controlled living conditions of the cohort may be considered a strength, as they reduce environmental confounding.

## 4. Materials and Methods

### 4.1. Participants

The study population comprised 95 residents of a long-term care facility (56 men, 59%; 39 women, 41%), with a mean age of 63.0 ± 14.0 years. Eligibility criteria included age ≥ 55 years and a minimum three-month stay in the institution. Exclusion criteria comprised the presence of acute infectious disease, active malignancy, and autoimmune disease. The selection of long-term-care facility residents provided a relatively homogeneous study population with respect to environmental exposure, dietary patterns, and daily routines, thereby reducing the influence of lifestyle-related confounders on the analyzed metabolic parameters.

All participants underwent a standard medical assessment, which included a clinical examination performed by an internist, anthropometric measurements, blood pressure evaluation, and routine laboratory tests. The study population was further divided into two groups according to the presence or absence of MetS, based on the 2022 Polish consensus criteria for MetS [2]. According to these criteria, MetS was diagnosed in participants presenting with central obesity (defined as BMI ≥ 30 kg/m^2^ or waist circumference ≥102 cm in men and ≥88 cm in women) in combination with at least two additional metabolic abnormalities, including elevated blood pressure or antihypertensive treatment, impaired glucose metabolism or glucose-lowering therapy, and atherogenic dyslipidemia or lipid-lowering treatment.

### 4.2. Anthropometric and Biochemical Assessments

Anthropometric measurements were conducted using standardized procedures. Body weight and height were measured under controlled conditions, and body mass index (BMI) was calculated as weight in kilograms divided by height in meters squared (kg/m^2^). Waist and hip circumferences were measured using a non-elastic measuring tape, and waist-to-hip ratio (WHR) was derived. Additional anthropometric parameters, including arm and thigh circumferences, were obtained according to a standardized protocol.

Biochemical analyses were performed in fasting venous serum samples. Concentrations of glucose, triglycerides (TGs), total cholesterol (TC), low-density lipoprotein cholesterol (LDL-C), and high-density lipoprotein cholesterol (HDL-C) were determined using standard enzymatic colorimetric methods according to the manufacturers’ instructions. Non-esterified fatty acids (NEFAs) were measured using a validated enzymatic colorimetric method.

The atherogenic index of plasma (AIP) was calculated as log10(TG/HDL-C) based on fasting serum lipid concentrations. All laboratory procedures were conducted under standardized conditions to ensure analytical accuracy and inter-assay reproducibility.

### 4.3. Genotyping

Genomic DNA was extracted from peripheral venous blood collected into EDTA-containing tubes using a commercial DNA isolation kit (Roche Diagnostics, Basel, Switzerland), according to the manufacturer’s instructions. DNA quality and concentration were assessed prior to analysis.

Genotyping of the VDR rs1544410 (BsmI, A/G) polymorphism was performed using a commercially available LightSNiP rs1544410 VDR Bsm assay (TIB MOLBIOL, Berlin, Germany) on a LightCycler^®^ 480 Real-Time PCR System (Roche Diagnostics, Basel, Switzerland). The assay contained sequence-specific primers and hybridization probes supplied by the manufacturer.

PCR amplification was performed in a final reaction volume of 20 μL containing 2.0 μL LightCycler^®^ 480 FastStart DNA Master HybProbe mix (Roche Diagnostics, Mannheim, Germany), 1.6 μL MgCl2 (25 mM), 1.0 μL reagent mix containing primers and probes, 1.0–5.0 μL genomic DNA (approximately 50 ng), and PCR-grade water to volume.

Thermal cycling conditions consisted of an initial denaturation step at 95 °C for 10 min, followed by 45 amplification cycles of 95 °C for 10 s, 60 °C for 10 s, and 72 °C for 15 s. Genotype discrimination was achieved by melting curve analysis performed at 95 °C for 30 s, 40 °C for 2 min, and gradual heating to 75 °C with continuous fluorescence acquisition.

Alleles were identified based on characteristic melting peaks corresponding to approximately 56.5 °C for the A allele and 66.3 °C for the G allele. Negative controls without template DNA were included in each run. To verify genotyping accuracy, a randomly selected subset of samples was re-genotyped, yielding 100% concordance.

### 4.4. Statistics

Biochemical and anthropometric parameters were expressed as mean ± standard deviation (SD). Categorical variables, including genotypes, were presented as frequencies and percentages.

Hardy–Weinberg equilibrium (HWE) was assessed by comparing observed and expected genotype frequencies using an online calculator (https://wpcalc.com/en/equilibrium-hardy-weinberg/, accessed on 16 March 2026).

The normality of distribution was evaluated using the Kolmogorov–Smirnov test. As several variables were not normally distributed, appropriate statistical tests were applied. Student’s *t*-test was used for normally distributed variables and the Mann–Whitney U test for non-normally distributed variables to compare biochemical and anthropometric parameters between MetS and non-MetS groups.

Homogeneity of variance was assessed using Levene’s test and was confirmed (*p* > 0.05).

The association between the rs1544410 (BsmI) VDR polymorphism, metabolic syndrome status, biochemical parameters, and anthropometric measurements was examined using two-way analysis of variance (ANOVA), including genotype, MetS status (MetS vs. non-MetS), and their interaction.

Sex was not included as a factor due to limited sample size.

Fisher’s LSD post hoc test was applied following a significant ANOVA result because the number of planned pairwise comparisons was limited, and this method provides greater statistical power for detecting subtle differences between groups compared with more conservative post hoc procedures.

The atherogenic index of plasma (AIP) was analyzed separately from the ANOVA model, as it is a derived measure based on TG and HDL-C concentrations. To avoid redundancy, AIP was assessed using multiple linear regression with dummy-coded rs1544410 genotypes, with the heterozygous genotype (AG) used as the reference category.

All statistical analyses were performed using STATISTICA 13 (TIBCO Software Inc., Palo Alto, CA, USA).

## 5. Conclusions

In conclusion, the rs1544410 polymorphism of the VDR gene was not associated with MetS status in institutionalized older adults but contributed to inter-individual variability in TG, NEFA, and AIP depending on metabolic status. These findings suggest that VDR-related genetic effects on lipid metabolism are context-specific and become more evident under metabolic stress conditions.

## Figures and Tables

**Table 1:** Characteristics of the study population.

Variable	Mean ± SD	Median (IQR)
Age (years)	62.7 ± 14.1	64 (54.0; 71.0)
SBP (mmHg)	122.04 ± 12.08	121.50 (113.00; 131.25)
DBP (mmHg)	76.53 ± 7.88	76.25 (72.25; 81.00)
Anthropometric parameters		
Body weight (kg)	76.4 ± 16.7	76 (63.7; 87.0)
Height (cm)	166.8 ± 9.8	165 (160.0; 175.0)
BMI (kg/m^2^)	27.5 ± 5.4	26.5 (23.5; 31.6)
Waist circumference (cm)	101.7 ± 15.1	102.0 (89.0; 113.0)
Arm circumference (cm)	29.2 ± 3.9	29.0 (26.0; 31.0)
Thigh circumference (cm)	49.3 ± 6.6	49.0 (44.0; 54.0)
Hip circumference (cm)	102.9 ± 10.1	103.0 (96.0; 108.0)
WHR	0.987 ± 0.098	1.0 (0.919; 1.056)
Biochemical parameters		
Glucose (mg/dL)	87.63 ± 33.51	76.29 (68.24; 97.91)
TG (mg/dL)	157.34 ± 68.29	139.63 (115.35; 169.83)
TC (mg/dL)	213.70 ± 51.59	202.59 (179.91; 239.71)
LDL-C (mg/dL)	106.01 ± 33.36	103.77 (89.62; 121.70)
HDL-C (mg/dL)	41.75 ± 13.04	39.22 (33.79; 46.47)
non-HDL-C (mg/dL)	171.94 ± 46.86	160.07 (145.56; 196.26)
NEFA (mmol/L)	0.929 ± 0.171	0.880 (0.818; 0.998)
AIP	0.207 ± 0.206	0.176 (0.060; 0.319)

Abbreviations: SBP—systolic blood pressure; DBP—diastolic blood pressure; BMI—body mass index; WHR—waist-to-hip ratio; TG—triglycerides; TC—total cholesterol; LDL-C—low-density lipoprotein cholesterol; HDL-C—high-density lipoprotein cholesterol; non-HDL-C—non-high-density lipoprotein cholesterol; NEFA—non-esterified fatty acids; AIP—atherogenic index of plasma; SD—standard deviation; IQR—interquartile range.

**Table 2:** Metabolic syndrome components and clinical characteristics.

Variable	Criterion (Cut-Off/Treatment)	n (%)
BMI	≥30 kg/m^2^	28 (29.5)
Waist circumference	≥102 cm (men), ≥88 cm (women)	59 (62.1)
Fasting glucose	≥100 mg/dL or glucose-lowering treatment	29 (30.5)
Non-HDL-C	≥130 mg/dL or lipid-lowering treatment	89 (93.7)
SBP/DBP	≥130/85 mmHg or antihypertensive treatment	53 (55.8)
Metabolic syndrome (MetS)	Obesity and 2 of 3 additional criteria	38 (40.0)

Abbreviations: BMI—body mass index; non-HDL-C—non-high-density lipoprotein cholesterol; SBP/DBP—systolic/diastolic blood pressure; MetS—metabolic syndrome.

**Table 3:** Clinical and biochemical characteristics of MetS and non-MetS participants.

Variable	MetS (n = 38)	Non-MetS (n = 57)	Test Statistic (*t* or U)	*p*
Age (years)	63.66 ± 15.93	62.07 ± 12.90	0.534	0.594
Body weight (kg)	79.30 ± 15.57	74.49 ± 17.21	1.384	0.170
Height (cm)	167.00 ± 10.00	166.00 ± 10.00	0.542	0.589
BMI (kg/m^2^)	28.22 ± 4.71	26.94 ± 5.86	1.124	0.264
Arm circumference (cm)	29.97 ± 3.42	28.74 ± 4.12	1.530	0.130
Thigh circumference (cm)	49.58 ± 6.06	49.14 ± 6.92	0.318	0.751
Waist circumference (cm)	104.79 ± 13.52	99.72 ± 15.78	1.622	0.108
Hip circumference (cm)	103.50 ± 7.80	102.54 ± 11.45	0.449	0.654
WHR (waist/hip ratio)	1.01 ± 0.11	0.97 ± 0.09	2.041	0.044
Glucose (mg/dL)	93.04 ± 42.05	84.01 ± 26.15	0.429	0.668
TG (mg/dL)	201.27 ± 85.45	128.06 ± 28.67	6.203	<0.001
TC (mg/dL)	219.95 ± 50.64	209.52 ± 52.23	0.964	0.337
LDL-C (mg/dL)	108.25 ± 25.65	104.52 ± 37.78	0.533	0.595
HDL-C (mg/dL)	34.17 ± 8.97	46.82 ± 12.92	−5.246	<0.001
NEFA (mmol/L)	0.97 ± 0.19	0.90 ± 0.15	1.641	0.101
AIP	0.40 ± 0.18	0.08 ± 0.09	8.223	<0.001

Abbreviations and notes: MetS—metabolic syndrome; non-MetS—no metabolic syndrome; WHR—waist-to-hip ratio; TG—triglycerides; TC—total cholesterol; LDL-C—low-density lipoprotein cholesterol; HDL-C—high-density lipoprotein cholesterol; NEFA—non-esterified fatty acids; AIP—atherogenic index of plasma. Student’s *t*-test was used for normally distributed variables, whereas the Mann–Whitney U test was applied for variables that did not meet the assumption of normality (TG, glucose, NEFA). A *p*-value < 0.05 was considered statistically significant.

**Table 4:** Hardy–Weinberg equilibrium for the rs1544410 (BsmI) polymorphism of the VDR gene.

Group	Genotype	Observed (Expected)	Allele Frequencies	χ^2^	*p*
MetS (n = 38)	GG	20 (19.2)	p(G) = 0.71; q(A) = 0.29	0.414	0.520
	AG	14 (15.6)			
	AA	4 (3.2)			
non-MetS (n = 57)	GG	26 (25.3)	p(G) = 0.67; q(A) = 0.33	0.158	0.691
	AG	24 (25.3)			
	AA	7 (6.3)			

Abbreviations and notes: MetS—metabolic syndrome; non-MetS—no metabolic syndrome. A *p*-value < 0.05 was considered statistically significant.

**Table 5:** Distribution of rs1544410 BsmI polymorphism in MetS and non-MetS participants.

Genotype/Allele	MetS (n = 38)	Non-MetS (n = 57)	χ^2^	*p*
Genotypes			0.450	0.451
GG–n (%)	20 (52.63)	26 (45.61)		
AG–n (%)	14 (36.84)	24 (42.11)		
AA–n (%)	4 (10.53)	7 (12.28)		
Alleles			0.406	0.524
G–n (%)	54 (71.05)	76 (66.67)		
A–n (%)	22 (28.95)	38 (33.33)		

Abbreviations and notes: MetS—metabolic syndrome; non-MetS—no metabolic syndrome. *p*-value < 0.05 was considered statistically significant.

**Table 6:** Effects of metabolic syndrome status and rs1544410 (BsmI) VDR polymorphism on anthropometric and biochemical parameters: two-way ANOVA.

Parameter	Group	Genotype			ANOVA			
		GG (n = 46)	AG (n = 38)	AA (n = 11)	Effect	F	*p*	η^2^
Body weight (kg)	MetS	75.70 ± 16.33	81.04 ± 15.21	91.20 ± 2.83	MetS	F(1,9) = 3.14	0.080	0.034
	non-MetS	76.65 ± 16.98	71.30 ± 18.22	77.41 ± 14.86	Genotype (BsmI)	F(2,89) = 1.10	0.338	0.024
					MetS × Genotype	F(2,89) = 1.46	0.239	0.032
Height (cm)	MetS	166 ± 10	168 ± 9	171 ± 13	MetS	F(1,89) = 0.62	0.433	0.007
	non-MetS	168 ± 9	164 ± 10	167 ± 13	Genotype (BsmI)	F(2,89) = 0.36	0.697	0.008
					MetS × Genotype	F(2,89) = 0.83	0.438	0.018
BMI (kg/m^2^)	MetS	27.23 ± 4.78	28.68 ± 4.52	31.52 ± 4.31	MetS	F(1,89) = 1.95	0.167	0.021
	non-MetS	27.21 ± 5.75	26.34 ± 6.13	28.02 ± 5.91	Genotype (BsmI)	F(2,89) = 0.92	0.403	0.020
					MetS × Genotype	F(2,89) = 0.68	0.511	0.015
Arm circumference (cm)	MetS	29.10 ± 3.58	30.64 ± 3.10	32.00 ± 2.94	MetS	F(1,89) = 2.32	0.131	0.025
	non-MetS	28.77 ± 3.74	28.29 ± 4.71	30.14 ± 3.48	Genotype (BsmI)	F(2,89) = 1.28	0.282	0.028
					MetS × Genotype	F(2,89) = 0.71	0.495	0.016
Thigh circumference (cm)	MetS	48.10 ± 5.48	50.29 ± 6.72	54.50 ± 4.20	MetS	F(1,89) = 1.86	0.176	0.020
	non-MetS	50.88 ± 6.77	47.75 ± 7.09	47.43 ± 6.19	Genotype (BsmI)	F(2,89) = 0.35	0.701	0.008
					MetS × Genotype	F(2,89) = 3.19	0.046	0.067
Waist circumference (cm)	MetS	101.60 ± 13.67	106.28 ± 13.71	115.50 ± 5.00	MetS	F(1,89) = 3.78	0.055	0.041
	non-MetS	100.65 ± 15.80	97.92 ± 16.34	102.43 ± 15.35	Genotype (BsmI)	F(2,89) = 1.15	0.320	0.025
					MetS × Genotype	F(2,89) = 1.01	0.368	0.022
Hip circumference (cm)	MetS	102.10 ± 8.67	105.79 ± 6.23	102.50 ± 8.19	MetS	F(1,89) = 0.09	0.768	0.001
	non-MetS	104.81 ± 10.83	99.83 ± 11.92	103.43 ± 11.79	Genotype (BsmI)	F(2,89) = 0.04	0.959	0.001
					MetS × Genotype	F(2,89) = 1.85	0.163	0.040
WHR (waist/hip ratio)	MetS	0.99 ± 0.09	1.00 ± 0.11	1.13 ± 0.13	MetS	F(1,89) = 7.92	0.006	0.082
	non-MetS	0.96 ± 0.09	0.98 ± 0.09	0.99 ± 0.09	Genotype (BsmI)	F(2,89) = 3.44	0.036	0.072
					MetS × Genotype	F(2,89) = 1.66	0.196	0.036
Glucose (mg/dL)	MetS	92.82 ± 36.04	84.44 ± 37.78	124.23 ± 75.84	MetS	F(1,89) = 3.33	0.072	0.036
	non-MetS	87.10 ± 28.85	79.41 ± 23.62	88.34 ± 25.04	Genotype (BsmI)	F(2,89) = 2.20	0.117	0.047
					MetS × Genotype	F(2,89) = 0.95	0.389	0.021
TG (mg/dL)	MetS	182.45 ± 45.71	181.05 ± 57.16	366.14 ± 148.91	MetS	F(1,89) = 93.27	<0.001	0.512
	non-MetS	131.27 ± 32.95	125.44 ± 25.50	125.08 ± 23.92	Genotype (BsmI)	F(2,89) = 16.96	<0.001	0.275
					MetS × Genotype	F(2,89) = 18.31	<0.001	0.291
TC (mg/dL)	MetS	208.89 ± 34.89	221.65 ± 57.44	269.27 ± 74.51	MetS	F(1,89) = 3.79	0.055	0.041
	non-MetS	221.66 ± 65.53	197.94 ± 38.66	204.20 ± 25.28	Genotype (BsmI)	F(2,89) = 1.11	0.331	0.025
					MetS × Genotype	F(2,89) = 2.97	0.056	0.063
LDL-C (mg/dL)	MetS	106.41 ± 24.40	111.46 ± 26.25	106.30 ± 35.79	MetS	F(1,89) = 0.68	0.410	0.008
	non-MetS	111.07 ± 43.97	101.68 ± 28.26	89.89 ± 41.68	Genotype (BsmI)	F(2,89) = 0.41	0.662	0.009
					MetS × Genotype	F(2,89) = 0.67	0.514	0.015
HDL-C (mg/dL)	MetS	34.34 ± 8.80	36.00 ± 8.95	26.85 ± 8.22	MetS	F(1,89) = 23.70	<0.001	0.210
	non-MetS	48.46 ± 15.74	44.91 ± 9.10	47.24 ± 13.50	Genotype (BsmI)	F(2,89) = 0.59	0.556	0.013
					MetS × Genotype	F(2,89) = 1.12	0.330	0.025
NEFA (mg/dL)	MetS	0.93 ± 0.15	0.94 ± 0.16	1.24 ± 0.32	MetS	F(1,89) = 9.52	0.003	0.097
	non-MetS	0.91 ± 0.19	0.89 ± 0.10	0.94 ± 0.15	Genotype (BsmI)	F(2,89) = 5.25	0.007	0.106
					MetS × Genotype	F(2,89) = 3.13	0.049	0.066

Abbreviations and notes: MetS—metabolic syndrome; non-MetS—no metabolic syndrome; BMI—body mass index; WHR—waist-to-hip ratio; TG—triglycerides; TC—total cholesterol; LDL-C—low-density lipoprotein cholesterol; HDL-C—high-density lipoprotein cholesterol; NEFA—non-esterified fatty acids. Two-way analysis of variance (ANOVA) was used to assess the effects of metabolic syndrome status (MetS vs. non-MetS), rs1544410 (BsmI) VDR genotype, and their interaction on the analyzed variables. F values represent main and interaction effects, and η^2^ indicates partial eta squared (effect size). Statistical significance was set at *p* < 0.05.

**Table 7:** Fisher’s LSD post hoc pairwise comparisons of rs1544410 (BsmI) genotype × metabolic syndrome status across metabolic and anthropometric parameters.

TG (mg/dL)						
	GG MetS	AG MetS	AA MetS	GG Non-MetS	AG Non-MetS	AA Non-MetS
GG MetS	-	0.931	<0.001	<0.001	<0.001	0.006
AG MetS		-	<0.001	0.002	<0.001	<0.001
AA MetS			-	<0.001	<0.001	<0.001
GG non-MetS				-	0.660	0.756
AG non-MetS					-	0.986
AA non-MetS						-
TC (mg/dL)						
	GG MetS	AG MetS	AA MetS	GG non-MetS	AG non-MetS	AA non-MetS
GG MetS	-	0.471	0.032	0.399	0.477	0.833
AG MetS		-	0.101	0.999	0.167	0.458
AA MetS			-	0.084	0.011	0.043
GG non-MetS				-	0.102	0.420
AG non-MetS					-	0.774
AA non-MetS						-
NEFA (mmol/L)						
	GG MetS	AG MetS	AA MetS	GG non-MetS	AG non-MetS	AA non-MetS
GG MetS	-	0.863	<0.001	0.580	0.375	0.921
AG MetS		-	0.001	0.498	0.329	0.971
AA MetS			-	<0.001	<0.001	0.003
GG non-MetS				-	0.712	0.625
AG non-MetS					-	0.467
AA non-MetS						-
Thigh circumference (cm)						
	GG MetS	AG MetS	AA MetS	GG non-MetS	AG non-MetS	AA non-MetS
GG MetS	-	0.336	0.075	0.152	0.859	0.814
AG MetS		-	0.255	0.781	0.248	0.344
AA MetS			-	0.302	0.057	0.085
GG non-MetS				-	0.091	0.214
AG non-MetS					-	0.908
AA non-MetS						-
WHR (ratio)						
	GG MetS	AG MetS	AA MetS	GG non-MetS	AG non-MetS	AA non-MetS
GG MetS	-	0.740	0.008	0.221	0.607	0.931
AG MetS		-	0.017	0.149	0.420	0.739
AA MetS			-	0.001	0.003	0.017
GG non-MetS				-	0.460	0.443
AG non-MetS					-	0.784
AA non-MetS						-

Abbreviations and notes: MetS—metabolic syndrome; non-MetS—no metabolic syndrome; TG—triglycerides; TC—total cholesterol; NEFA—non-esterified fatty acids; WHR—waist-to-hip ratio. Values represent *p*-values from pairwise post hoc comparisons (LSD test) within a two-way ANOVA model including rs1544410 (BsmI) genotype and metabolic syndrome status (MetS vs. non-MetS). The table presents all pairwise genotype × metabolic status comparisons. Statistical significance was set at *p* < 0.05.

## Data Availability

The BsmI data presented in this study are available from the corresponding author upon reasonable request. The data are not publicly available due to privacy concerns.

## References

[1] Alberti K.G., Eckel R.H., Grundy S.M., Zimmet P.Z., Cleeman J.I., Donato K.A., Fruchart J.C., James W.P., Loria C.M., Smith S.C. (2009). International Diabetes Federation Task Force on Epidemiology and Prevention; Hational Heart, Lung, and Blood Institute; American Heart Association; World Heart Federation; International Atherosclerosis Society; International Association for the Study of Obesity. Harmonizing the metabolic syndrome: A joint interim statement of the International Diabetes Federation Task Force on Epidemiology and Prevention; National Heart, Lung, and Blood Institute; American Heart Association; World Heart Federation; International Atherosclerosis Society; and International Association for the Study of Obesity. Circulation.

[2] Dobrowolski P., Prejbisz A., Kuryłowicz A., Baska A., Burchardt P., Chlebus K., Dzida G., Jankowski P., Jaroszewicz J., Jaworski P. (2022). Metabolic Syndrome—A New Definition and Management Guidelines: A Joint Position Paper by the Polish Society of Hypertension, Polish Society for the Treatment of Obesity, Polish Lipid Association, Polish Association for Study of Liver, Polish Society of Family Medicine, Polish Society of Lifestyle Medicine, Division of Prevention and Epidemiology, Polish Cardiac Society, “Club 30” Polish Cardiac Society, and Division of Metabolic and Bariatric Surgery, Society of Polish Surgeons. Arch. Med. Sci..

[3] McCarthy K., O’Halloran A.M., Fallon P., Kenny R.A., McCrory C. (2023). Metabolic Syndrome Accelerates Epigenetic Ageing in Older Adults: Findings from the Irish Longitudinal Study on Ageing (TILDA). Exp. Gerontol..

[4] Chen M.Z., Wong M.W.K., Lim J.Y., Merchant R.A. (2021). Frailty and Quality of Life in Older Adults with Metabolic Syndrome—Findings from the Healthy Older People Everyday (HOPE) Study. J. Nutr. Health Aging.

[5] Goddard G., Rajagopal S., Wahbah Makhoul G., Raji M.A. (2025). Metabolic Syndrome in Older Adults: Through the Lens of Institute for Healthcare Improvement’s (IHI) 4Ms Framework and Social Determinants of Health. Life.

[6] Sales M.C.S., Oliveira L.P., Liberalino L.C.P., Cunha A.T.O., Sousa S.E.S., Lemos T.M.A.M., Lima S.C.V.C., Costa Lima K., Sena-Evangelista K.C.M., Pedrosa L.F.C. (2018). Frequency of metabolic syndrome and associated factors in institutionalized elderly individuals. Clin. Interv. Aging.

[7] Kočar E., Šket R., Halužan Vasle A., Avguštin G., Benedik E., Koroušić Seljak B., Simić P., Martinko A., Morrison S.A., Sorić M. (2025). Measuring biological age: Insights from omics studies. Ageing Res. Rev..

[8] Vitezova A., Zillikens M.C., van Herpt T.T., Sijbrands E.J., Hofman A., Uitterlinden A.G., Franco O.H., Kiefte-de Jong J.C. (2015). Vitamin D Status and Metabolic Syndrome in the Elderly: The Rotterdam Study. Eur. J. Endocrinol..

[9] Yun H., Jang K. (2026). Association between Vitamin D Deficiency and Cardiometabolic Risk Clustering among Rural Community-Dwelling Older Adults: A Cross-Sectional Study. Healthcare.

[10] Liu L., Cao Z., Lu F., Liu Y., Lv Y., Qu Y., Gu H., Li C., Cai J., Ji S. (2020). Vitamin D Deficiency and Metabolic Syndrome in Elderly Chinese Individuals: Evidence from the CLHLS. Nutr. Metab..

[11] Zhao Y., Su D., Huang L., He M., Han D., Zhao D., Zou Y., Zhang R. (2025). Prevalence of Metabolic Syndrome with Different Serum Vitamin D Levels in Middle-Aged and Older Adults. Nutr. Metab..

[12] Carlberg C. (2003). Current Understanding of the Function of the Nuclear Vitamin D Receptor in Response to Its Natural and Synthetic Ligands. Recent Results Cancer Res..

[13] Zmijewski M.A., Carlberg C. (2020). Vitamin D Receptor(s): In the Nucleus but Also at Membranes?. Exp. Dermatol..

[14] Bischoff-Ferrari H.A., Borchers M., Gudat F., Dürmüller U., Stähelin H.B., Dick W. (2004). Vitamin D Receptor Expression in Human Muscle Tissue Decreases with Age. J. Bone Miner. Res..

[15] Giustina A., Bouillon R., Dawson-Hughes B., Ebeling P.R., Lazaretti-Castro M., Lips P., Marcocci C., Bilezikian J.P. (2023). Vitamin D in the Older Population: A Consensus Statement. Endocrine.

[16] Fronczek M., Osadnik T., Banach M. (2023). Impact of Vitamin D Receptor Polymorphisms in Selected Metabolic Disorders. Curr. Opin. Clin. Nutr. Metab. Care.

[17] Totonchi H., Rezaei R., Noori S., Azarpira N., Mokarram P., Imani D. (2021). Vitamin D Receptor Gene Polymorphisms and the Risk of Metabolic Syndrome (MetS): A Meta-Analysis. Endocr. Metab. Immune Disord. Drug Targets.

[18] Filus A., Trzmiel A., Kuliczkowska-Płaksej J., Tworowska U., Jędrzejuk D., Milewicz A., Medraś M. (2008). Relationship between Vitamin D Receptor BsmI and FokI Polymorphisms and Anthropometric and Biochemical Parameters Describing Metabolic Syndrome. Aging Male.

[19] Tourkochristou E., Mouzaki A., Triantos C. (2023). Gene Polymorphisms and Biological Effects of the Vitamin D Receptor in Nonalcoholic Fatty Liver Disease Development and Progression. Int. J. Mol. Sci..

[20] Alhawari H., Jarrar Y., Abulebdah D., Abaalkhail S.J., Alkhalili M., Alkhalili S., Alhawari H., Momani M., Obeidat M.N., Fram R.K. (2022). Effects of Vitamin D Receptor Genotype on Lipid Profiles and Retinopathy Risk in Type 2 Diabetes Patients: A Pilot Study. J. Pers. Med..

[21] Guzel Dirim M., Hasanoglu Sayin S., Senkal N., Tuncel F.C., Oyaci Y., Medetalibeyoglu A., Kose M., Pehlivan S., Tukek T. (2026). Vitamin D Receptor Gene Variations and Their Association with Cardiometabolic Risk and Microvascular Complications in Metabolic Dysfunction-Associated Steatotic Liver Disease: Evidence from a Turkish Cohort. Lab. Med..

[22] Schuch N.J., Garcia V.C., Vívolo S.R., Martini L.A. (2013). Relationship between Vitamin D Receptor Gene Polymorphisms and the Components of Metabolic Syndrome. Nutr. J..

[23] Contreras-Bolívar V., García-Fontana B., García-Fontana C., Muñoz-Torres M. (2021). Mechanisms Involved in the Relationship between Vitamin D and Insulin Resistance: Impact on Clinical Practice. Nutrients.

[24] Szymczak-Pajor I., Drzewoski J., Śliwińska A. (2020). The Molecular Mechanisms by Which Vitamin D Prevents Insulin Resistance and Associated Disorders. Int. J. Mol. Sci..

[25] Haussler M.R., Whitfield G.K., Haussler C.A., Hsieh J.C., Thompson P.D., Selznick S.H., Dominguez C.E., Jurutka P.W. (1998). The Nuclear Vitamin D Receptor: Biological and Molecular Regulatory Properties Revealed. J. Bone Miner. Res..

[26] Uitterlinden A.G., Fang Y., van Meurs J.B.J., Pols H.A.P., van Leeuwen J.P.T.M. (2004). Genetics and Biology of Vitamin D Receptor Polymorphisms. Gene.

[27] Czyżniewski B., Chmielowiec J., Pruszyńska-Oszmałek E., Hajduk-Warchoł M., Chmielowiec K., Kołodziejski P., Huzarski T., Checińska-Maciejewska Z., Kowalski M.T., Szczap E. (2025). Associations between Vitamin D2 and Metabolic Indices in Institutionalized Older Adults: A Cross-Sectional Study. J. Physiol. Pharmacol..

[28] Rahmadhani R., Zaharan N.L., Mohamed Z., Moy F.M., Jalaludin M.Y. (2017). The Associations between VDR BsmI Polymorphisms and Risk of Vitamin D Deficiency, Obesity and Insulin Resistance in Adolescents Residing in a Tropical Country. PLoS ONE.

[29] Maaruf S.M., Mohammad D.K., Hassan T.S., Aziz A.D., Yashooa R.K., Hassan H.T., Agha S.S.F., Daham R.N.A., Aali M.H., Mustafa S.A. (2026). Association of VDR BsmI Polymorphism and Vitamin D Status with Osteoarthritis Susceptibility. BMC Med. Genom..

[30] Divanoglou N., Komninou D., Stea E.A., Argiriou A., Papatzikas G., Tsakalof A., Pazaitou-Panayiotou K., Georgakis M.K., Petridou E. (2021). Association of Vitamin D Receptor Gene Polymorphisms with Serum Vitamin D Levels in a Greek Rural Population (Velestino Study). Lifestyle Genom..

[31] Sinha N., Bhattacharya A., Deshmukh P.R., Panja T.K., Yasmin S., Arlappa N. (2016). Metabolic Syndrome among Elderly Care-Home Residents in Southern India: A Cross-Sectional Study. WHO South East Asia J. Public Health.

[32] Ataei S.M., Sheidaei A., Golestani A., Khosravi S., Rashidi M.M., Tabatabaei-Malazy O., Haghshenas R., Khalagi K., Larijani B. (2025). Over-Time Changes in the Prevalence of Metabolic Syndrome and Its Components among Elderly Population in Iran from 2016 to 2021: A Nationwide Study. PLoS ONE.

[33] Tune J.D., Goodwill A.G., Sassoon D.J., Mather K.J. (2017). Cardiovascular Consequences of Metabolic Syndrome. Transl. Res..

[34] Dobiášová M. (2006). AIP—Atherogenic Index of Plasma as a Significant Predictor of Cardiovascular Risk: From Research to Practice. Vnitr. Lek..

[35] Ulloque-Badaracco J.R., Hernandez-Bustamante E.A., Alarcon-Braga E.A., Mosquera-Rojas M.D., Campos-Aspajo A., Salazar-Valdivia F.E., Valdez-Cornejo V.A., Benites-Zapata V.A., Herrera-Añazco P., Valenzuela-Rodríguez G. (2022). Atherogenic Index of Plasma and Coronary Artery Disease: A Systematic Review. Open Med..

[36] Tworowska-Bardzińska U., Lwow F., Kubicka E., Łaczmański Ł., Jędrzejuk D., Dunajska K., Milewicz A. (2008). The Vitamin D Receptor Gene BsmI Polymorphism Is Not Associated with Anthropometric and Biochemical Parameters Describing Metabolic Syndrome in Postmenopausal Women. Gynecol. Endocrinol..

[37] Khan S.M., El Hajj Chehadeh S., Abdulrahman M., Osman W., Al Safar H. (2018). Establishing a Genetic Link between FTO and VDR Gene Polymorphisms and Obesity in the Emirati Population. BMC Med. Genet..

[38] Hajj A., Chedid R., Chouery E., Megarbané A., Gannagé-Yared M.H. (2016). Relationship between Vitamin D Receptor Gene Polymorphisms, Cardiovascular Risk Factors and Adiponectin in a Healthy Young Population. Pharmacogenomics.

